# Toxic Picoplanktonic Cyanobacteria—Review

**DOI:** 10.3390/md13031497

**Published:** 2015-03-18

**Authors:** Natalia Jakubowska, Elżbieta Szeląg-Wasielewska

**Affiliations:** Department of Water Protection, Faculty of Biology, Adam Mickiewicz University in Poznań, Umultowska 89, 61-614 Poznań, Poland; E-Mail: eszelag@amu.edu.pl

**Keywords:** picocyanobacteria, cyanotoxins, picoplankton, toxicity

## Abstract

Cyanobacteria of a picoplanktonic cell size (0.2 to 2.0 µm) are common organisms of both freshwater and marine ecosystems. However, due to their small size and relatively short study history, picoplanktonic cyanobacteria, in contrast to the microplanktonic cyanobacteria, still remains a poorly studied fraction of plankton. So far, only little information on picocyanobacteria toxicity has been reported, while the number of reports concerning their presence in ecosystems is increasing. Thus, the issue of picocyanobacteria toxicity needs more researchers’ attention and interest. In this report, we present information on the current knowledge concerning the picocyanobacteria toxicity, as well as their harmfulness and problems they can cause.

## 1. Introduction

Picoplankton is the smallest size fraction of plankton. The size of organisms of picoplanktonic cell size ranges from 0.2 to 2.0 µm [1]. The group contains hetero- and autotrophic organisms. The latter group contains the following groups: Chlorophyta, Bacillariophyta, and Picobiliphytes [2,3,4], as well as prokaryotic Cyanobacteria [5,6,7,8].

Autotrophic picoplankton (APP) is a part of aquatic ecosystems that is still poorly studied. In the past, it has been confused with heterotrophic bacteria (HB). In 1968, Bailey-Watts *et al.* [9] for the first time confirmed the previous presumptions of Holmes and Anderson [10] concerning the existence of APP. Nevertheless, research on this group has developed very slowly, but the use of more sophisticated techniques, like epifluorescence microscopy [11], electron microscopy [2], flow cytometry [12] and other molecular biology methods have expanded current knowledge of APP.

It is already known that picocyanobacteria, like heterotrophic bacteria, constitute an important part of the microbiological loop [13,14]. They incorporate the dissolved organic matter (DOM) into the food web [15]. Thus, they comprise an extremely important element of aquatic ecosystem function [16,17,18]. Furthermore, they usually have relatively low biomass [19], high growth rate and a high amount of pressure from consumers [8,20,21]. Due to their small size picocyanobacteria comprise the main food source of the nanoplanktonic protozoans: Ciliata, Flagellata and larger zooplankton [22,23,24,25]. As reported by Stockner [3] and Sorokin *et al.* [26], picoplankton can constitute up to 98% of biomass and phytoplankton production. As picoplankton comprise such a high proportion of phytoplankton, they significantly influence not only the rate and the amount of the matter but also the energy that flows to higher trophic levels [15,27]. In the surface layers of water, the number of picocyanobacteria ranges from a few to even a few hundred thousand [28,29] and, at times, even to a few million cells in 1 mL of water [27,30,31,32]. The high number and metabolic activity of APP and HB clearly influences the ecological stability of water ecosystems [18,33]. Still, detailed studies on the picocyanobacteria physiology are lacking. Understanding this group is even more important considering that some species are members of the potentially toxic Cyanobacteria, which can create blooms and release cyanotoxins.

## 2. Cyanobacterial Blooms

From early spring to late summer, when the water temperature ranges from 15 to 30 °C, cyanobacteria may create blooms. The blooms (*i.e.*, the massive occurrence of cyanobacteria) are accompanied by water turbidity and color changes [34,35]. The blooms result mainly in decreased biodiversity as a consequence of the dominance of one or, at most, a few species [35].

The first reports that are potentially consistent with mass blooms of cyanobacteria were recorded almost 2000 years ago, during the military campaigns of General Zhu Ge-Ling in southern China [36]. The next accounts dates to the 12th century from southwestern Scotland, in the vicinity of the former *Monasterium Virdis Stagni* (Monastery of the Green Loch), located near the Soulseat Loch, 2 miles from Stranraer [37]. Records of blooms that harmed people were not reported until 1793 in Canada [38] and 1842 in Great Britain [39]. Numerous incidences of death and illnesses of farm animals and dead fishes were reported in 1878 in Australia, and in Poland in 1884 [40]. Regularly increasing numbers of reports of cyanobacteria blooms and intoxications caused by cyanotoxins have motivated the creation of numerous documentations, statistics and analyses in order to better understand the phenomenon.

The most frequently observed blooms are connected with progressive eutrophication of aquatic ecosystems or other such disruptions [41]. The growth and development of cyanobacteria in inland and marine waters depends on their ability to gain food resources and exploit them with the lowest losses. That is why the increased availability of nutrients encourages blooms of cyanobacteria [35,42]. Moreover, numerous adaptations and habitat preferences are correlated with the size of cyanobacteria cells. Studies conducted since the 1980s show that cyanobacteria occur at a similar quantity in reservoirs with high as well as low trophic states [43,44,45].

## 3. Morphology and Physiology of Picocyanobacteria

In natural environments, picocyanobacteria exhibit dramatic seasonal fluctuations [8,19,46,47] in which the species composition as well as the number and form of their occurrence change [46,48,49,50]. Picocyanobacteria can occur as single cells or as microcolonies with thickly or loosely arranged cells [30,47,50,51,52]. In marine waters, single cells are most common and are represented by species of the genus *Prochlorococcus* and *Synechococcus*. However, freshwater picocyanobacteria diversity is much greater. Previous studies of inland reservoirs have shown that picocyanobacteria density is highest in the summer. At that time, the proportion of picocyanobacteria occurring in the colony forms increases [50,53]; this pattern is found in oligo-, meso-, and eutrophic reservoirs [28,54]. Although there are hypotheses suggesting that aggregations occur as a result of genetics, survival mechanisms, pressure from consumers, or unfavorable external conditions, such as nutrient depletion, the exact nature of this phenomenon is still unknown [50,55].

The distribution of these organisms in the pelagic zone is also variable [56,57] and influenced by temperature (1–30 °C) and light tolerance [55,58]. Light that favors the development of picocyanobacteria falls into the range from 45 to 2000 μmol photon m^−2^ s^−1^ [59,60]. Light within this range permits picocyanobacteria to live in deeper water levels, with a lower light intensity found at the base of the euphotic layer. In this water zone green light does not exceed 1% of the radiation that reaches the surface of water [56,61,62].

Picocyanobacteria are photoautotrophs that, beside chlorophyll “a”, also contain accessory pigments, such as phycocyanin and phycoerythrin, while their predominant carotenoid is zeaxanthin [63,64]. The abovementioned phycobiliproteins, including allophycocyanin, absorb the green and blue–green, through yellow–orange to red light, which significantly exceeds the spectrum of absorbance of chlorophyll “a” [65]. Thus, the cells of picocyanobacteria have an increased ability to absorb the photons and use them in photosynthesis. Moreover, a decreased intensity of light does not change the assembly of carbon atoms into photosynthetic products. The ability to survive and regrow after periods of being in full darkness gives picocyanobacteria an advantage over larger algae.

Picocyanobacteria are also more able to assimilate biophilic compounds; this is possible because of the thin layer of “still” water around individual cells that results in a favorable (*i.e.*, high) ratio of cell membrane area to cell volume [66]. The supplementation of nitrogen deficiencies from phycocyanin contained within their cells, as well as a higher rate of intake of nutrients, give these organisms a higher chance under nitrogen limitation [67,68]. This is also enabled by the wide spectrum of their occurrence in aquatic ecosystems. In contrast to some microplanktonic cyanobacteria considered as indicators of eutrophication, picocyanobacteria occur commonly in waters of all trophic types [43]. Picocyanobacteria are even found in oligotrophic ecosystems, where they are especially important to primary production.

## 4. Harmfulness and Toxicity

A combination of pigments, small size slowing down sedimentation and active usage of nutrients allows picocyanobacteria to grow intensively at great depths for many months. It must be emphasized that the phenomenon of creating an over-surface maximum concentration of chlorophyll can be a huge threat for the water intake located in an area of picoplankton development [56]. Furthermore, these organisms can secrete a number of secondary metabolites, however, many of these compounds may have useful properties, e.g., antibacterial, antiviral, anticoagulant or anticancer [69].

A major category of cyanobacterial secondary metabolites is oligopeptides, which includes a range of both proteinogenic and non-proteinogenic amino acids. Oligopeptides have highly variable structure and many of these compounds share common conserved substructures [69]. These structural similarities are used to classify oligopeptides into seven major peptide classes: aeruginosins [70], anabaenopeptins [71], cyanopeptolins [72], cyclamides [73], microcystins [74], microginins [75] and microviridins [76].

Many of these oligopeptides are synthesized through non-ribosomal pathways; although ribosomal synthesis, coupled with further posttranslational modifications has also been documented in the production of a few oligopeptide classes. Unlike ribosomal products, non-ribosomal oligopeptides are assembled by large multifunctional enzyme complexes, such as NRPS (non-ribosomal peptide synthetase) or NRPS/PKS (polyketide synthases) hybrid pathways. These pathways can produce particular structures that are not achievable by ribosomal peptide synthesis. NRPS and PKS genes often, and unexpectedly, occur within a single open reading frame [77].

Recently, cyanobacteria have been found to produce bioactive compounds through ribosomal pathways [78]. Ribosomally synthesized and post-translationally modified peptides are produced by a pathway referred to as post-ribosomal peptide synthesis [79]. These metabolites called cyanobactins, and they contain heterocyclized amino acids or isoprenoid amino acid derivatives [80]. Moreover, cyanobactins contain oxazolines, thiazolines or their oxidized derivatives, oxazoles, thiazoles and cyclic peptides, which consist solely of proteinogenic amino acids [81]. Currently, more than a hundred cyanobactins have been identified. The biosynthetic genes underlying cyanobactin production have been described in a many genera of cyanobacteria, including picoplanktonic *Prochlorococcus* and *Synechococcus* [82].

Picocyanobacteria, including few strains of *Synechococcus* as well as *Synechocystis*, and *Aphanocapsa*, synthesise 2-methylisoborneol (MIB) and geosmin (1,2,7,7-tetramethyl-2-norborneol) (GSM), secondary matabolites that are also taste and odor compounds produced as secondary metabolites [83,84,85]. MIB is produced and released throughout the life cycle of cyanobacteria, whilst geosmin is trapped in the cell bodies and released in high concentrations when individual cells die. These organic compounds can cause problems, especially in drinking water, because the human taste and odor detection thresholds for these compounds are between 5 and 10 ng L^−1^ [86]. However, reports note concentrations of picocyanobacteria reaching even 100 ng L^−1^, as observed in Lake Bowen [85].

A potential threat also results from the fact that picocyanobacteria are members of families in which toxic strains occur. Geosmin and MIB frequently co-occur with cyanotoxins in lakes and reservoirs, though most species of cyanobacteria are not capable of producing taste and odor compounds and cyanotoxins simultaneously [87]. It has to be expected, however, that they can also secrete toxic metabolites that are subject to bioaccumulation and biomagnification [88,89]. Due to that issue, they may have negative influences on other links of the food chain, and thus people [36,90,91].

Known toxins secreted by cyanobacteria can be divided into four basic groups, relating to their functionality: hepatotoxins, neurotoxins, cytotoxins and dermatotoxins [89,92,93]. These compounds are not considered to have significant roles in processes of metabolism and growth. It is claimed that secondary metabolites are produced as a chemical defense against predators of higher trophic levels or of cyanobacteria competition for resources with sympatric organisms [94,95,96]. Toxins can also play a role as signal substances or be a relict of evolution [38]. Chronic or sporadic contact with cyanotoxins can, however, cause pathological symptoms that may have either additive or synergistic effects.

As previously mentioned, cyanotoxins are compounds coded by clusters of genes that are responsible for their synthesis and transportation, as well as the synthesis of other enhancing enzymes. Biosynthesis of microcystins, a type of hepatotoxin, begins with transcription of the *mcy cluster* which spans 55 kb and is comprised of 10 genes arranged in two independently transcribed operons, *mcyA*-C and *mcyD*-J. NRPS, PKS and other enzymes are encoded by the larger operon, while the smaller *mcyA*-*C* encodes three NRPSs [97]. Other cyanotoxins are less well known yet are probably encoded in a similar manner. Biosynthesis of cylindrospermopsin (cytotoxin) is encoded by three predicted genes responsible for the following proteins: aoaA-aminotransferase, aoaB-NRPS, and aoaC-PKS. The gene encoding aoaB spans 5606 bp and is adjacent to aoaA, which is 1179 bp in length. The *aoaC* gene is transcribed in an opposite direction and spans 4299 bp [98]. Such neurotoxins are only now being studied, which makes it difficult to determine the function of consecutive genes. As reported by Mejean *et al.* [99], anatoxin is encoded in the *ana cluster*, comprising *anaA*-D and *anaE*-H, and probably synthesising PKS.

NRPS genes have been confirmed in 75% of 146 axenic cyanobacterial strains (including *Cyanocystis* PCC 9504) from 35 genera and five orders (Chroococcales, Pleurocapsales, *Oscillatoriales*, Nostocales and Stigonematales). However, those genes were not detected in *Cyanothece*, *Synechococcus* and *Synechocystis* strains [100]. Studies that attempt to detect of cyanotoxin biosynthesis genes in picocyanobacteria are lacking. However, physical-chemical studies based on the chromatographic analyses of water samples where picocyanobacteria were noted, compounds of such type were observed. It has also been shown that within one species of cyanobacteria, both toxic and non-toxic strains may occur [101,102,103,104]. It is also important to note that picoplanktonic cyanobacteria are a relatively difficult group of organisms for laboratory analyses. Many species cannot be maintained in cultures (so called VBNC–Viable but Nonculturable) similar to heterotrophic bacteria [105,106]. This makes gaining better knowledge of them and verifying the assumptions regarding their secretion of toxic metabolites difficult.

### 4.1. Hepatotoxins

Hepatotoxins are the group of cyanotoxins that occur most commonly. Microcystins and nodularins are classified into this group. Microcystins are cyclic heptapeptides, formed non-ribosomally in the cytoplasm with molecular weights from 900 to 1100 Da and a general formula of cyclo-(d-Ala1-X2-d-MeAsp3-Z4-Adda5-d-glutamate6-Mdha7) [107,108,109], while d-MeAsp3 is a d-erythro-β-methylaspartic acid. Adda is toxic and characteristic only for cyanobacteria acids containing 20 atoms of carbon (2S,3S,8S,9S)-3-amino-9-methoxy-2,6,8-trimethyl-10-phenyldeca-4,6-dienoic, and Mdha is *N*-methyldehydroalanine. X and Z indicate changing residues of l amino acids, such as leucine (L), arginine (R), alanine (A), tyrosine (Y), methionine (M), tryptophan (W), phenylalanine (F). These one-letter symbols of the residues of L amino acids are used in nomenclature of consecutive analogues of microcystins, about 90 of which are already known [77]. Every analogue is characterized by a different toxicity. The lethal dose for mice of the most commonly found and the most toxic Microcystin-LR (MC-LR) is 50 μg kg^−1^ while, for example, the MC-RR form has a lethal dose of 500–800 μg kg^−1^ [108].

The effect of microcystins is based on the specific transport of amphipathic organic compounds occurring in the hepatocytes’ cellular membranes. It consists of non-covalent bonds of serine/threonine phosphatases PP1 and PP2A in the hepatocytes’ cytosol. This causes inhibition of the phosphatases’ activity and, as a result, excessive phosphorylation of peptides (intermediate filaments and microfilaments), and also damage to the cytoskeleton of hepatocytes [110]. Damage to liver caused by microcystins can also occur as a result of an extended level of γ-glutamyl transferase [111,112].

Nodularins act analogically and similarly to microcystins LR are cyclo(-d-MeAsp1-l-Arg2-Adda3-d-Glu4-Mdhb5), where Mdhb stands for 2-(methylamino)-2(*Z*)-dehydrobutiric acid. Currently, 11 analogues of this toxin were found differing in regard to presence and absence of methyl groups in positions 1, 3 and 5 [89,113,114]. Dose LD_50_ (for mice) is 50 μg kg^−1^ [115].

In the initial phase of blooms, cyanotoxins occur inside the cyanobacteria cells. Decaying of the blooming, thus cell lysis, causes the release of toxic metabolites that leads to the increase of their concentration in the water. The presence of microcystins is noted in 50% to 90% of samples taken during blooming and their concentrations reach even 25 mg dm^−3^ of water and 20 mg in one gram of dry matter [108]. Nodularins, like microcystins, are highly soluble in water; they are not, however, as common. They were found most often in seawater, in which a marine species occurs, *Nodularia spumigena* Mertens ex Bornet and Flahault 1888 [89].

Hepatotoxins bioaccumulate and biomagnify in subsequent links of the food chain. They were found in tissues of planktonic crustaceans, snails, larvae of crabs, bivalves and fishes [36,90,116,117,118,119,120,121,122]. In cases of intoxications, internal bleeding and liver damage can occur. Characteristic symptoms of intoxications are gastrointestinal and liver disorders, weakness and anorexia. Hepatotoxins induce apoptosis and necrosis of hepatocytes and are also promoters of cancerous processes [123].

Hepatotoxin secretion by picoplanktonic species was first reported in 1999; these compounds were first isolated from six strains of picocyanobacteria collected in northern Brazil. Four of these strains were identified as colonial *Aphanocapsa cumulus*, Komárek and Cronberg 1999. The other two strains formed loosely dispersed cells. Picocyanobacteria were grown under controlled laboratory conditions, during which toxicity tests were performed. An insensitive HPLC analysis showed the presence of MC-LR in just one sample of single cells, whereas ELISA assays confirmed the presence of toxins in all cultures. Concentrations of microcystins there were very low (undetectable by HPLC) ranging from 0.08 to 3.7 ng mg^−1^ [124]. Water was collected from waste stabilization pond (WSP) in Cajati, São Paulo State, from which five species of picocyanobacteria were isolated, from the genus *Merismopedia.* ELISA assays detected a concentration of microcystins in these *Merismopedia* sp. CENA106 cultures of 2.17 μg L^−1^ [125]. Bláha and Maršálek [126] confirmed toxicity in the following cultures: *Synechococcus nidulans* (Pringsheim), Komárek in Bourrelly 1970 strain CCALA 188 (=UTEX 625); *Cyanobium rubescens* (T.P.Chang), Komárek, Kopeck and Cepák 1999 strain SAG 381 (isol. Chang 1979); and *Cyanobacterium cedrorum* (Sauvageau), Komárek, Kopecky and Cepák 1999 strain CCAP 1479/2a (SAG 88.79). HPLC analysis of the first two strains identicated that they poduce MC-LR; *C. cedrorum* was the most toxic. The HPLC analysis also detected other unidentified hepato- and cytotoxic compounds (*i.e.*, compounds lacking standards). Later reports identified the secretion of MC-LR and MC-YR by marine *Synechococcus* isolated from the Salton Sea, California (strain SS-1) [127]. From 1999 to 2000, *Synechococcus* sp. SS-1, was one of the dominant organisms that formed harmful algal blooms. In 2009–2011 marine *Synechococcus*, as well as *Aphanothece parallelliformis* Cronberg 2004; *Aphanocapsa delicatissima* W. West and G.S. West 1912; *Aphanocapsa incerta* (Lemmermann) Cronberg and Komárek 1994; *Cyanodictyon planctonicum* Meyer 1994; *Cyanodictyon imperfectum* Cronberg and Weibull 1981; and *Merismopedia tenuissima* Lemmermann 1898, in 2009–2011 were noted in the Baltic Sea. The concentration of nodularin measured by HPLC and LC-MS/MS in environmental samples was noted then at a level of 0.1–2.2 μg L^−1^ [128]. However, studies confirming secretion of nodularin by the picoplanktonic species are missing.

### 4.2. Neurotoxins

Neurotoxins are a group of alkaloids containing anatoxin-a, with derivatives, anatoxin-a (s), and saxitoxin with analogues and non-proteinous amino acid β-Methylamino-l-alanine (BMAA) [38,129]. Concerning the chemical structure, anatoxin-a is an alkaloid with a molecular mass of 165 Da, LD_50_ ranging from 200 to 250 μg kg^−1^ and a general formula of 2-acetyl-9-azabicyclo[4.2.1]non-2-ene [130], while homoanatoxin-a is its analogue with a molecular mass of 179 Da, and it is a (2-(propan-1-oxo-1-yl)-9-azabicyclo[4.2.1]non-2-ene [131,132]. Anatoxin-a and its analogues are strong agonists of the nicotinic cholinergic receptor occurring in the postsynaptic membrane. Their action leads to an opening of the ionic channels and permanent depolarization of the neuromuscular synapse. Toxins bind with the acetylcholine receptor but can be hydrolysed by acetylcholinesterase, resulting in an over-stimulation of muscle cells [133,134,135]. Shortly after intoxication with anatoxins, the following symptoms occur: trembling and contraction of muscles, balance disturbance, abdominal troubles, and, in extreme cases, death from asphyxiation as a result of paralysis of the respiratory muscles [38].

Anatoxin-a (s), with a molecular mass of 252 Da and LD_50_ of 20 μg kg^−1^, is a unique phosphate ester of a cyclic *N*-hydroxyguanine, inhibiting acetylcholinesterase activity [36,136,137,138]. The mechanism of its activity is the quasi-irreversible inhibition of the enzyme acetylcholine-ACh, which causes neurological disorders similar to those caused by organophosphorous and carbamate insecticides (parathion and malathion) [38,138,139]. Except for salivation being the basic symptom of the activity of anatoxin-a (s), lack of ACh hydrolysis and opening of the ionic channels causes dysfunction of muscles by their exhaustion, which can lead even to death of the organism [139,140].

Saxitoxin, also called paralytic shellfish toxins (PSTs), as well as its thirty other isomers, are heterocyclic guanidinium compounds, that inhibit the functional potential along the nervous appendage as a result of the blockage of the transport of Na^+^ ions through the sodium channels in nervous cells [141,142]. Except for the neurological activity causing motor disruptions, involuntary muscles palsy and paralysis, saxitoxins also show dermotoxic and cytotoxic effects, such as skin rash, eye irritation, abdominal pains and symptoms similar to influenza [38,143]. Intraperitoneal dose LD_50_ for mice is 5 μg kg^−1^, while the lethal dose for humans is 1 mg kg^−1^ [142].

BMAA with a molecular mass of 118.13 Da [144] causes disruption in the tertiary structure of proteins and their activity; it also disrupts function of the neuronal receptors and is an antagonist of glutamate [38,145,146,147]. The effect of its action is onset of amyotrophic lateral sclerosis/Parkinsonism dementia complex (ALS/PDC) [147,148,149,150], manifesting as memory deficits, olfactory deficits, disorientation, personality changes, muscle weakness or atrophy, bradykinesia and gait disturbance [150,151]. The lethal dose LD_50_ for mice is 301 mg kg^−1^, while for larger mammals it is 1043 mg kg^−1^ [152]. As shown by studies of a terrestrial ecosystem of Guam (Pacific Ocean, Mariana Islands) [153], an inland aquatic ecosystem in Nebraska [144] and laboratory studies of *Apis mellifera* and other species [150], BMAA undergoes bioaccumulation and is a “slow toxin”. In the organism BMAA occurs mainly in a form bounded to proteins creating an inner reservoir of the compound, from which a slow release of the neurotoxins during the metabolism of proteins causes neuro-pathological interactions.

Previous reports confirm the synthesis of BMAA by two species of picocyanobacteria. Secretion of BMAA by the marine strain *Prochlorococcus marinus* CCMP1377, isolated from the Sargasso Sea was observed at a level of 32 μg g^−1^ Free BMAA and 57 μg g^−1^ Protein BMAA [148]. In the case of freshwater *Synechococcus* PCC 6301, the toxicity was lower (25 μg g^−1^ Free BMAA, with no detectable Protein BMAA) [148]. However, no other neurotoxins have been found to be synthesized by picocyanobacteria.

### 4.3. Cytotoxins

Cytotoxins are a group of toxins that includes many NRPS compounds (e.g., aeruginosin, acutiphicin, ambigol A and B, apratoxin A, curacin A, cyanobacterine, hectochlorin and dolastatin 10, nostocyclamide, patellamide A, raocyclamide A and B, scytoficine, tubercidine and wewakazole) [154,155] and two ribosomal peptides (microcyclamides and aerucyclamides) [156]. However, mitsoamide and cylindrospermopsin are the most common. Mitsoamide is a linear lipopeptide that was isolated from the marine cyanobacterium *Geitlerinema* sp. [157]. Cylindrospermopsin is a compound that contains tricyclic guanidine moiety combined with a hydroxymethyl uracil [158]. It is a “slow toxin”, with LD_50_ of 2.1 mg kg^−1^ (after 24 h) and LD_50_ of 0.2 mg kg^−1^ (after 5–6 days). Cylindrospermopsin has several paths of toxic activity: inhibition of glutathione, synthesis of proteins and cytochrome P450 [159], oxidative stress, leading to loss of chromosomes and splitting DNA [160,161], and disruptions of the cell cycle as a result of which apoptosis or necrosis of cells occur [162]. Cylindrospermopsin causes liver and kidneys damage as well as pathological changes of lungs, heart, stomach, adrenal glands and of the vascular and lymphatic systems [162,163]. Cytotoxins are secreted by cyanobacteria species from the orders Nostocales and Oscillatoriales.

At this time, the information on the possibility of secretion of cytotoxic metabolites by picoplanktonic cyanobacteria is lacking.

### 4.4. Dermotoxins

Dermotoxins are a group of liposacharids and indole alkaloids to which lyngbyatoxins, aplysiatoxin and debromo aplysiatoxins belong. Three isoforms of lyngbyatoxins can be distinguished: lyngbyatoxin-a, -b, and -c [164] with a molecular mass of 437 Da [165]. These are compounds with LD_50_ (for mice) of 250 μg kg^−1^ [166]. Due to their lipophilic properties, they cause skin irritations, such as skin itching, skin redness, skin burning, skin blistering and skin swelling [165]. Intake of lyngbyatoxins leads to the inflammations of oesophagus and alimentary canals [164]. Similar symptoms of activity are also observed following exposure to aplysiotoxins and debromo aplysiatoxins. These are, however, phenolic bislactones of 671 Da and 592 Da, with LD_50_ in mice of 100–120 μg kg^−1^ [167].

Lipopolisacharids (LPS) are the compounds of the external cell wall of gram-negative bacteria and cyanobacteria, including picoplanktonic *Synechococcus* and *Synechocystis* [107,168,169]. They have an external protective layer that protects them against unfavorable environmental factors, preventing lysis by complements, antimicrobial peptides and detergents [170]. Studies of heterotrophic bacteria have shown that LPS is comprised of four covalently linked segments: a surface carbohydrate polymer (*O*-specific chain); a core oligosaccharide, featuring an outer and inner region (core R); and an acylated glycolipid (lipid A) [171]. However, the LPS structure of *Synechococcus* is slightly different; They lack heptose and Kdo is replaced by 4-linked glucose-in the oligosaccharide core, and they contain a lipid A moiety uniquely characterized by odd-chain hydroxylated fatty acids and a lack of phosphates [170]. According to Takada and Kotani [172], this probably influences toxic activity of cyanobacterial LPS that is almost five times lower than LPS of *E. coli* [173]. LD_50_ of the cyanobacterial LPS (in mice) ranges from 40 to 190 mg kg^−1^ and depends on the cyanobacteria species [171].

Liposacharids have strongly allergic and irritating effects. They cause skin and eye irritation as well as other allergic reactions. After intake they can also cause symptoms typical for influenza: subfebrile body temperature, shivers, headaches, nausea, muscle and joints aches, drowsiness, mild amnesia and diarrhea [171]. According to Best *et al.* [174], LPS also causes decreased activity of glutathione *S*-transferases (GSTs), which participate in detoxification of many xenobiotics. Decreased availability of GSTs can also be harmful to the ability of the organisms to detoxify microcystins.

### 4.5. Other Toxic Compounds

Physical-chemical studies conducted on *Synechococcus* sp. BP-1 have found that they have the ability to synthesize a unique sulfolipid with thioic *O*-acid ester (thionsulfolipid). The structure of this thionsulfolipid was identified as 6-sulfo-α-d-quinovopyranosyl- (1→1′)-2′-*O*-acyl-3′-*O*-thioacyl-d-glycerol. Furthermore it has been shown that the compound has toxic properties on HL 60 human lymphoma cells. After 24-h exposure of HL to thionsulfolipid, at a concentration of 200 μg mL^−1^, an inhibition of growth of 61% of cells was observed. Moreover, a test conducted on minnows found an LD_50_ of 20 mg kg^−1^ [175].

It is certain that a metabolic pathway for alkylthiocarbonyl exists because alkylthioic O-acid ester formation frequires alkylthiocarbonyl. This compound has not yet been found in any other photosynthetic organisms. It is assumed that alkylthioic *O*-acid ester is more efficient in photosynthesis than fatty acid esters or it may be a vestige of the incipient lipid metabolism [175].

## 5. Conclusions

Picocyanobacteria, despite their ubiquity, are a group of organisms still relatively poorly known. The basis of the monitoring studies that prove their presence in freshwater reservoirs is increasing, however, and knowledge of their toxicity is still scarce. The lack of complex studies of individual species, or even genera is striking. Few reports regarding the secretion of microcystins, neurotoxins or LPS by picocyanobacteria have been published (Table 1). The few published so far are mainly field studies, in which the explicit determination of the cyanotoxins’ sources is lacking. By far, behavioral characteristics of these organisms still comprise a limitation in monocultural studies as some of these species are VBNC. However, taking into consideration the development of new techniques and analytical possibilities, the chances of increasing knowledge of picocyanobacteria physiology are getting more likely.

It is necessary to take note of the role of these organisms in aquatic ecosystems, especially since they occur in all trophic types of water. Thus, their presence can be highly hazardous to people and other organisms using water intakes that are predominated by picocyanobacteria.

**Table 1:** Comparison of potentially toxic species of picocyanobacteria.

Genus	Species/Strain	Toxin	Source
*Synechocystis*	*Synechocystis*	LPS	[86,108,127,168,169]
		Microcystin	
		BMAA	
	*Synechocystis aqualitis*	Microcystin LR	[124]
	*Synechocystis* (SyncWTP97)	Microcystin	[176]
*Synechococcus*	*Synechococcus nidulans* CCALA 188	Microcystin	[126]
	*Synechococcus*	LPS	[86,108,127,128,168,169,177]
		Microcystin	
		BMAA	
		Nodularin	
	*Synechococcus* PCC 6301	BMAA	[148]
	*Synechococcus* SS-1	Microcystin	[127]
	*Synechococcus* BP-1	Thionsulfolipid	[175]
	*Synechococcus* CENA 108	Microcystin	[125]
*Cyanodictyon*	*Cyanodictyon planctonicum*	Nodularin	[128]
	*Cyanodictyon imperfectum*	Nodularin	[128]
*Cyanobium*	*Cyanobium rubescens* SAG 381	Microcystin	[126]
*Cyanobacterium*	*Cyanobacterium cedrorum* CCAP 1479/2a	Microcystin	[126]
*Aphanocapsa*	*Aphanocapsa cumulus*	Microcystin LR	[124,178]
	*Aphanocapsa delicatissima*	Nodularin	[128]
	*Aphanocapsa incerta*	Nodularin	[128]
	*Aphanocapsa* sp.	LPS Microcystin	[86]
*Aphanothece*	*Aphanothece stratus*	Nodularin	[178]
	*Aphanothece parallelliformis*		[128]
*Merismopedia*	*Merismopedia* CENA106	Microcystin	[125]
	*Merismopedia tenuissima*	Nodularin	[128]
*Romeria*	*Romeria carauru*	Microcystin	[178]
*Prochlorococcus*	*Prochlorococcus marinus* CCMP1377	BMAA	[148]
